# Dynamic changes in the body composition during chemotherapy for gastrointestinal tumors in the context of active nutrition intervention

**DOI:** 10.3389/fonc.2022.965848

**Published:** 2022-11-29

**Authors:** Ting Xu, Zhenhao Li, Hui Li, Jixiang Hou, Jingjing Li, Gaowa Jin, Shaohua Li, Quanfu Li

**Affiliations:** ^1^ Ordos Clinical College, Inner Mongolia Medical University, Ordos, China; ^2^ School of Public Health and Management, WenZhou Medical University, Zhejiang, China; ^3^ Department of Medical Oncology, Ordos Central Hospital, Ordos, China; ^4^ Department of Paediatrics, Ordos Maternal and Child Health Hospital, Ordos, China

**Keywords:** nutritional intervention, skeletal muscle, dynamic changes, malignant tumor, chemotherapy

## Abstract

**Objective:**

To explore the dynamic changes in the body composition during chemotherapy in patients with gastrointestinal malignancies in the context of active nutrition intervention.

**Methods:**

Patients with gastrointestinal malignancies receiving first-line chemotherapy in the Department of Medical Oncology of Ordos Central Hospital from September 2019 to January 2022 were included in this study. The Nutritional Risk Screening form 2002, Patient-Generated Subjective Global Assessment form, bioelectrical impedance analysis, and dynamic changes in L3 skeletal muscle index (SMI) (L3SMI) were assessed at baseline and after chemotherapy. The recommended protocol of the *Nutrition Guidelines for Cancer Patients in China 2020* was adopted as the active nutrition intervention. Chemotherapy-related toxic adverse reactions and the degree of toxicity were recorded with the adoption of the *Common Terminology Criteria for Adverse Events* version 4.0 by the National Institutes of Health. The type of toxicity Chemotherapy-Induced Nauseaand Vomiting(CINV) and hematological.

**Results:**

Fifty cases were enrolled in the study, and 38 cases completed the dynamic follow-ups. The average follow-up time was 125.63 d. In the context of active nutrition intervention, the prevalence of sarcopenia decreased from 26.3% before chemotherapy to 21.1% after chemotherapy. The average L3SMI decreased from 38.77 cm^2^/m^2^ to 38.04 cm^2^/m^2^, with a reduction of 1.41% ± 8.49% (*P* = 0.177). The SMI remained stable or increased in 57.9% (22/38) of patients. The benefit of active nutrition intervention was greater in the sarcopenic group than in the non-sarcopenic group (*P* = 0.033). There was an increased incidence of chemotherapy-related toxic adverse reactions of ≥ grade 3 during chemotherapy in the sarcopenic group compared with the muscle retention/gain group (*P* = 0.089).

**Conclusion:**

Active nutrition intervention might decrease the degree of reduction of L3SMI and the incidence of sarcopenia in patients with gastrointestinal tumors and raise the proportion of patients with stable or increased SMI during chemotherapy.

## 1 Introduction

Muscle wasting is a major nutritional problem in patients with cancer due to the cancer-mediated inflammatory response that promotes catabolism, as evidenced by decreased protein synthesis, increased muscle catabolism, and hypermetabolism. Skeletal muscle loss is present in >50% of patients who are diagnosed with malignancy for the first time (1, 2).

Over the past 10 years, sarcopenia has been a new direction in the field of oncology nutrition. Bioelectrical impedance analysis (BIA), dual-energy X-ray absorptiometry, and computed tomography (CT) scanning are the three most widely used methods for body composition measurement. Unlike the first two measurements, CT measures the body composition at the tissue–organ level and can accurately quantify tissue area, volume, and attenuation, and is considered the gold standard for body composition assessment. In healthy individuals, the L3 skeletal muscle area (L3SMA) has been shown to correlate well with whole-body muscle (3). In patients with cancer, CT is routinely adopted to diagnose and monitor disease progression. Therefore, CT can be used to measure L3SMA and further calculate the L3 skeletal muscle index (SMI) (L3SMI) to assess the body composition of patients.

Using L3SMI as a representative body composition analysis method, patients with solid tumors (such as gastric cancer, pancreatic cancer, and colorectal cancer) with sarcopenia have been shown to have a higher incidence of toxic reactions and a worse prognosis in chemotherapy (4–7). Previous studies focused on the correlation between skeletal muscle quantity and quality at baseline and treatment-related toxic reactions as well as the prognosis (4). Since 2015, more attention has been paid to the relationship between the dynamic changes in skeletal muscle and adverse reactions, efficacy, and prognosis before and after treatment, with 60% of investigations on the dynamic changes in skeletal muscle during treatment being published after 2018 (8). It was found that the baseline nutritional status of patients with nasopharyngeal carcinoma was not correlated with prognosis, and a decrease in nutritional status during radiotherapy suggested a poor prognosis (9). Investigations on sarcopenia are developing from static to dynamic monitoring, which is more in line with the needs of clinical oncology nutrition.

Gastrointestinal malignancies have the most significant impact on human nutritional status due to their special physiological functions. A meta-analysis showed the L3SMI of patients with solid tumors decreased during chemoradiotherapy, especially with the most obvious decrease being in digestive system malignancies, such as esophageal cancer. The decrease in the muscle index of patients with advanced gastric cancer receiving chemotherapy indicated a poor prognosis (8, 10). Currently, reports in this field are mainly from retrospective analyses of European and American populations, and to reduce confounding factors, none of these studies included patients who received active nutrition interventions.

Therefore, a prospective clinical investigation was conducted in northern China to compare the dynamic changes in the body composition of patients with gastrointestinal malignancies during chemotherapy in the context of active nutrition intervention. The relationship with chemotherapy-related toxic reactions was also analyzed.

## 2 Materials and methods

A prospective, self-control study was conducted to investigate the dynamic changes in L3SMI in patients with gastrointestinal malignancies admitted to the Department of Medical Oncology of Ordos Central Hospital from September 2019 to January 2021 who received six cycles of chemotherapy. The present study was approved by the medical ethics committee of the hospital (2021-013). All enrolled patients signed an informed consent form. The study was registered in the Chinese Clinical Trial Registry (ChiCTR2200056758).

### 2.1 The inclusion and exclusion criteria

The inclusion criteria were as follows: (1) patients aged ≥18 years old with pathologically confirmed malignancy to be treated with chemotherapy; (2) patients with a Karnofsky Performance Status score of ≥70 points; (3) patients with no abnormality in the hepatic and renal functions and a normal electrocardiogram; (4) a pre-chemotherapy blood test in which the white blood cell count > 3.5 × 10^9^/L, neutrophil count > 1.5 × 10^9^/L, platelet count > 85 × 10^9^/L, alkaline phosphatase ≤ 2.5 × the upper limit of the normal reference range, serum alanine transferase and aspartate aminotransferase ≤ 3 × the upper limit of the normal reference range, bilirubin ≤ 1.5 × the upper limit of the normal reference range, and creatinine ≤ 1.5 × the upper limit of the normal reference range; (5) patients without other diseases of the liver, kidney, or blood system; (6) patients without relevant contraindications to chemotherapy; (7) patients who did not have interventions (e.g. surgery) to change the body composition between CT scanning and the initiation of chemotherapy; (8) patients who were eligible for regular BIA and CT follow-ups; (9) patients who undertook adjuvant or first-line chemotherapy, or those with second-line chemotherapy with an interval of >12 months; and (10) patients who gave informed consent and signed the consent form.

The exclusion criteria were as follows: (1) patients with contraindications to chemotherapy; (2) patients who were bedridden at the time of enrollment and could not cooperate with the nutritional assessment; (3) patients who were comorbid with other serious wasting diseases; (4) patients with a history of long-term hormone administration; (5) patients who had HIV, active hepatitis, kidney disease, or other diseases that affected drug excretion or interfered with drug metabolism between the combined medications; (6) patients who discontinued the antitumor therapy on their own or failed to follow up with body composition analysis or adverse reactions as scheduled; and (7) patients who had the administration of long-acting leukocyte-raising drugs prophylactically before chemotherapy.

### 2.2 Active nutrition intervention

Nutrition education was conducted by specialists in the department or fulltime nutrition educators at least twice a week during the hospitalization, and by designated senior physicians or fulltime nutrition educators at least once a week during the outpatient clinic. An online patient education session conducted by famous domestic tumor nutrition experts was organized every two months to help patients correct their misconceptions about tumor nutrition (11). The energy intake in the patients was assessed using the Brief Rating Scale by Cong Minghua (12).

The recommended methods in the *Nutrition Guidelines for Cancer Patients in China* issued by the China Cancer Nutrition and Supportive Treatment Committee of the Chinese Anti-Cancer Association were followed (13). The criteria for energy and protein intake in the 2020 version stated that the patient’s daily intake (including diet + enteral and/or parenteral nutrition) should reach at least 70% of the target level. The total energy requirement was 25–30 kcal/kg/d, where the weight was the ideal weight calculated according to the patient’s height. The protein requirement was 1.0–2.0 g/kg/d, which was calculated using the same weight.

A five-step approach for malnutrition was followed in the nutritional support as follows: (1) diet + nutrition education, (2) diet + oral nutrition supplement, (3) total enteral nutrition support, (4) partial enteral nutrition + partial parenteral nutrition support, and (5) total parenteral nutrition support. When the next step failed to meet 60% of the target requirement for over 3–5 d, the previous step was chosen for nutrition therapy.

The indications for enteral nutrition were patients with moderate to severe swallowing obstruction, weight loss of ≥5% within one month, and body mass index (BMI) of<18.5 kg/m^2^, with the Patient-Generated Subjective Global Assessment (PG-SGA) score of ≥4, with<60% of normal food intake for over 3–5 d, and still with digestive and absorption functions. The enteral nutrition supplementation involved oral nutritional support given to each patient within the range of 400–600 ml/d, which was dynamically adjusted by the clinician and dietitian. The adjustment was based on the changes in the nutritional status (especially weight), swallowing obstruction, swallowing pain, food intake, and diet structure. The adjustment included the route of enteral nutrition, nutritional requirements, and the composition ratio of nutrients. In addition, the consideration of whether to give tube-feeding enteral nutrition or parenteral nutrition support was decided in combination with the patient’s wishes.

**Figure 1:**
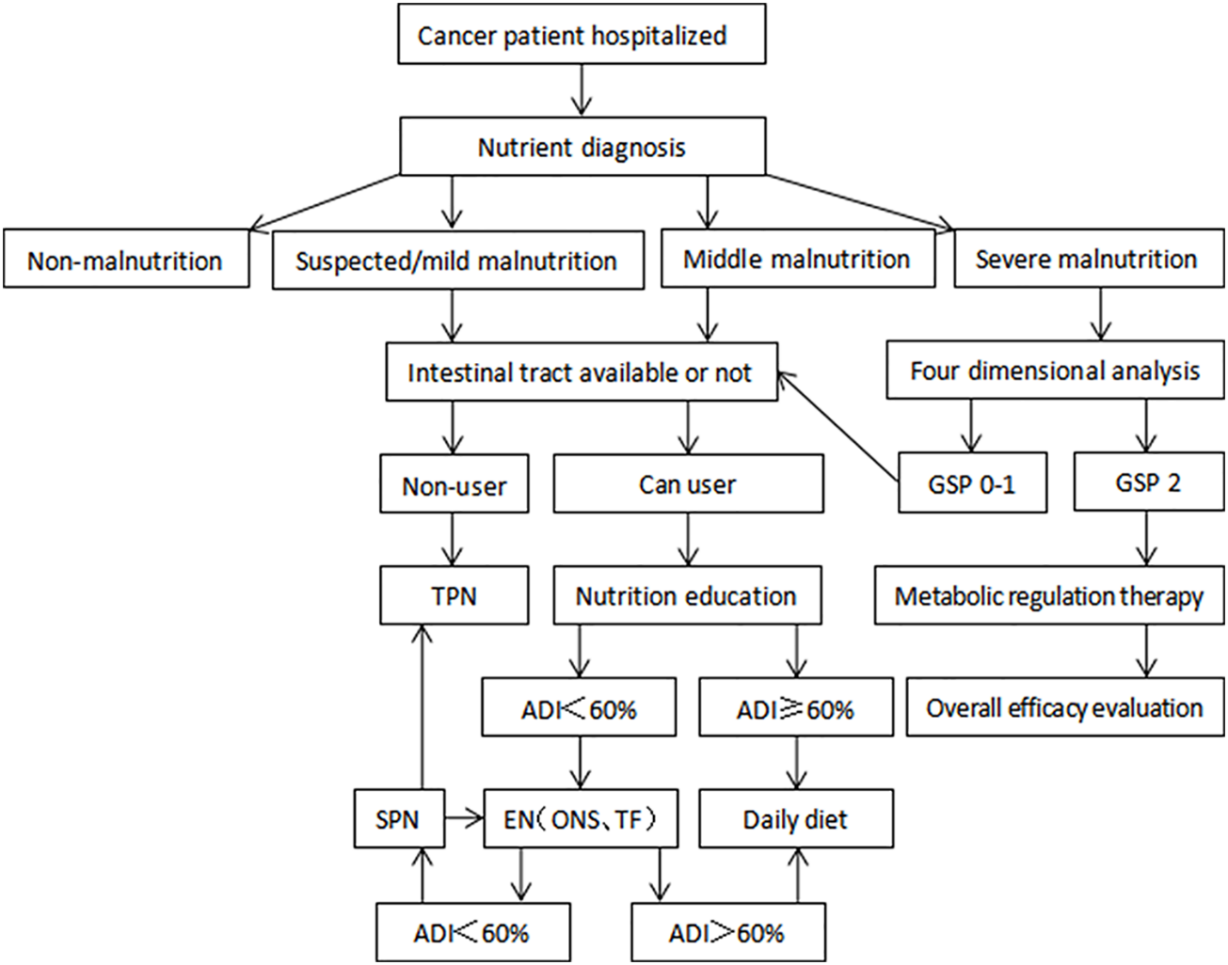
The flow chart of nutrition therapy for patients with cancer.

**Figure 1** represents the flow chart of nutrition therapy for patients with cancer according to the *Nutrition Guidelines for Cancer Patients in China 2020 (* 13).

### 2.3 Data collection

#### 2.3.1 General information collection

General information (including name, gender, age, home town, contact information, disease type, pathological stage, and initial chemotherapy regimen) was collected from all study subjects upon the first admission.

#### 2.3.2 Height and weight measurement

Height and weight measurements were completed according to standard methods at each admission for each patient and the BMI, together with body surface area (BSA), were calculated with the formulas:


$$\text{BMI = Weight(kg) }÷{\text{ Height}}^{2}(m^{2})$$

$${\text{BSA(m}}^{2}\text{) = (Height[cm] + Weight[kg] - 60) }÷100$$

#### 2.3.3 Body composition measurement

The following tests were performed, and the data were recorded within 30 d of baseline and within 30 d of the completion of total chemotherapy:

(1) The nutritional risk screening (NRS) 2002 and PG-SGA assessment: All study subjects were assessed by professionals on these scales within 48 h of admission, and all patients at nutritional risk were provided with active, positive, and rigorous nutrition education and support.

(2) The BIA measurement: Body fat composition, skeletal muscle, and the fat percentage (FP) of all enrolled patients were measured with a multi-frequency bioelectrical impedance analyzer (DBA-550). For this, it was necessary to maintain a suitable ambient temperature (20°C–25°C). The measurement was conducted after fasting, and the patient was required to accomplish defecation and urination before the measurement. The measurement should be avoided after exercise, showering, and during menstruation. The calculation of the fat-free mass index (FFMI) is:


$$\text{FFMI = (1 - FP) }\times \text{ Weight(kg) }÷{\text{ Height(m}}^{2})$$

The lower limits of FFMI were as follows: FFMI ≤17.4kg/m^2^ in males and FFMI ≤15.0kg/m^2^ in females (13).

(3) Abdominal CT scanning: After the enrollment and before chemotherapy, the abdominal CT of the lumbar 3 vertebral body was continuously sliced, with −29 to +150HU units and a slice thickness of 5 mm. The images of two consecutive transverse slices of the same sequence were selected to analyze the muscle area, and the radiotherapy TPS software XiO was used to calculate the sum of the cross-sectional area of the skeletal muscle (including the Psoas major, erector spinae, quadratus lumborum, transverse abdominis, external oblique, and internal oblique). The formulas are (14):


$${\text{L3SMI = L3SMA(cm}}^{2}\text{) }÷{\text{ Height}}^{2}(cm^{2})$$

$$\text{Lean body mass (LBM) = 0}.3\times {\text{ L3SMA(cm}}^{2}) + 6.06$$

#### 2.3.4 Laboratory tests and follow-up of toxic adverse reactions

Fasting venous blood was collected from patients within 24 h after each admission and the test results were recorded, including hepatic function, renal function, and peripheral blood routine test. Hematological and non-hematological toxic adverse reactions were followed up dynamically during the chemotherapy, and adverse reactions were graded and recorded according to the *Common Terminology Criteria for Adverse Events* version 4.0 (15).

### 2.4 Statistical methods

The SPSS™ Statistics v26.0 software was used for data analysis. The measurement data that satisfied the normal distribution were expressed as means ± standard deviation (x ± s), and the Wilcoxon signed rank–sum test and the Mann–Whitney U-test were used for other data. The one-sample *t*-test was used for one-way analysis of data, the paired-sample *t*-test was used for the analysis of data with changes before and after the intervention, and the independent-sample *t*-test was used for comparison between groups for a certain indicator. Pearson’s test and the Chi-squared test were used for correlation analysis. The countable data with frequency distribution were expressed as rates. A value of *P*< 0.05 was considered statistically significant.

## 3 Results

### 3.1 The clinical characteristics of the patients

In the present study, 50 patients were enrolled, and 38 cases (73.7% male) completed the dynamic follow-ups. The clinical characteristics of the enrolled patients with gastrointestinal malignancies are summarized in **Table 1**. The average age of the patients was 60.84 ± 11.84 years, and the follow-up time was 125.63 ± 43.69 d. The evaluation criteria for sarcopenia in Asian patients with cancer were L3SMI<36 cm^2^/m^2^ (male) and L3SMI<29 cm^2^/m^2^ (female) (16).

**Table 1:** Clinical characteristics of patients.

Characteristics	Sarcopenia (N=10) (A)	Non-Sarcopenia (N=28) (B)	Total (N=38)
**Age, Year**	63.30 ± 7.32	59.96 ± 13.08	60.84 ± 11.84
**Follow-up time,Day**	122.80 ± 62.09	126.64 ± 36.43	125.63 ± 43.69
**Gender**			
Man	7 (70.0%)	21 (75.0%)	28 (73.7%)
Female	3 (30.0%)	7 (25.0%)	10 (26.3%)
**Tumour location**			
Oesophagus & Stomach	9 (90.0%)	19 (67.9%)	28 (73.7%)
Pancreas	1 (10.0%)	3 (10.7%)	4 (10.5%)
Colon & Rectum	0 (0%)	6 (21.4%)	6 (15.8%)
**Type of chemotherapy**			
Adjuvant chemotherapy	5 (50.0%)	20 (71.4%)	25 (65.8%)
Palliative chemotherapy	5 (50.0%)	8 (28.6%)	13 (34.2%)
**Chemotherapy regimen**			
SOX/XELOX/FOLFOX6	8 (80.0%)	21 (75.0%)	29 (76.3%)
Others	2 (20.0%)	7 (25.0%)	9 (23.7%)
**Nutritional active intervention**			
Nutritional tube	4 (40.0%)	3 (10.7%)	7 (18.4%)
Nutrition education	6 (60.0%)	25 (89.3%)	31 (81.6%)
**Toxicity reaction (grade≥3)**			
Baseline status	6 (60.0%)	4 (14.3%)	10 (26.3%)
Whole process	10 (100%)	15 (53.6%)	25 (65.8%)
**Body mass index (kg/m^2^)**			
<18.5 Underweight	5 (50.0%)	1 (3.6%)	6 (15.8%)
18.5-23.9 Normal	5 (50.0%)	16 (57.1%)	21 (55.3%)
24.0-27.9 Overweight	0 (0%)	11 (39.3%)	11 (28.9%)

All patients were divided into the sarcopenic group (Group A) and the non-sarcopenic group (Group B). The BMI grouping method referred to the criteria in the *Nutrition Guidelines for Cancer Patients in China 2020 (* 13), in which BMI<18.5 was regarded as low body weight (malnutrition), BMI in the range of 18.5–23.9 was regarded as normal, and BMI in the range of 24–27.9 was regarded as overweight. The incidence of sarcopenia was 26.3% (10/38). All patients received active nutrition interventions, of whom 81.6% received nutrition education and 18.4% received gastrointestinal nutrition tube support. In the sarcopenic group, the incidence of chemotherapy-related toxic adverse reactions of ≥ grade 3 was significantly higher than in the non-sarcopenic group at baseline (60.0% vs. 14.3%) and throughout chemotherapy (100% vs. 53.6%). In the sarcopenia group, 10 patients were recorded, 4 with hematological toxicity (3 gastroesophageal cancer and 1 pancreatic cancer), and 6 with CINV (all were gastroesophageal cancer).

### 3.2 Changes in the body composition-related indicators before and after chemotherapy

All patients were divided into the chemotherapy baseline group (Group G1) and the chemotherapy completion group (Group G2). The changes in BMI, stem-loop 3 (SL3), L3SMI, LBM, FFMI, the PG-SGA scale, and the NRS 2002 scale in all patients before and after chemotherapy are summarized in **Table 2**. The PG-SGA score was significantly lower after chemotherapy compared with that before chemotherapy in the context of active nutrition intervention (*P* = 0.038). Although the NRS 2002 score did not show a significant difference after chemotherapy (*P* = 0.151), a decreasing trend occurred. Although SL3, L3SMI, and LBM were reduced by 1.44% ± 8.48%, 1.41% ± 8.49%, and 1.27% ± 7.04%, respectively, after chemotherapy, the differences were not statistically significant (*P* = 0.168, *P* = 0.177, and *P* = 0.168, respectively). The FFMI measured by BIA increased by 0.69% ± 5.28% after chemotherapy (*P* = 0.516), with an increase of 0.47 ± 5.01% in patients with colorectal cancer (17.68 ± 1.86 vs. 17.70 ± 1.29, *P* = 0.952), and FP increased by 0.82 ± 11.47% after chemotherapy (29.50 ± 5.34 vs. 30.12 ± 6.59, *P* = 0.660).

**Table 2:** Comparison of body composition related indicators between G1 and G2.

	G1	G2	Percent change (%)	P
**BMI (kg/m^2^)**	22.00 ± 3.45	21.80 ± 3.31	-0.66 ± 5.92	0.343
<18.5	16.77 ± 0.88	17.10 ± 0.47	2.18 ± 6.21	0.475
18.5-23.9	21.35 ± 1.67	21.07 ± 1.81	-1.18 ± 6.03	0.340
24.0-27.9	26.10 ± 1.48	25.75 ± 1.54	-1.20 ± 5.64	0.448
**SL3 (cm^2^)**	110.65 ± 25.48	108.45 ± 23.49	-1.44 ± 8.48	0.168
**L3SMI (cm^2^/m^2^)**	38.77 ± 7.04	38.04 ± 6.55	-1.41 ± 8.49	0.177
**LBM(kg)**	39.26 ± 7.64	38.60 ± 7.05	-1.27 ± 7.04	0.168
**FFMI (kg/m^2^)**	16.94 ± 1.77	17.05 ± 1.76	0.69 ± 5.28	0.516
**PG-SGA**	8 (6.75,12.25)	6 (4,10)		0.038
**NRS 2002**	4 (3,5.25)	3 (2,4.25)		0.151

BMI, body mass index; SL3, skeletal muscle area of the third lumbar spine; L3SMI, L3 skeletal muscle index; LBM, lean body mass; FFMI, fat-free mass index.

**Figure 2:**
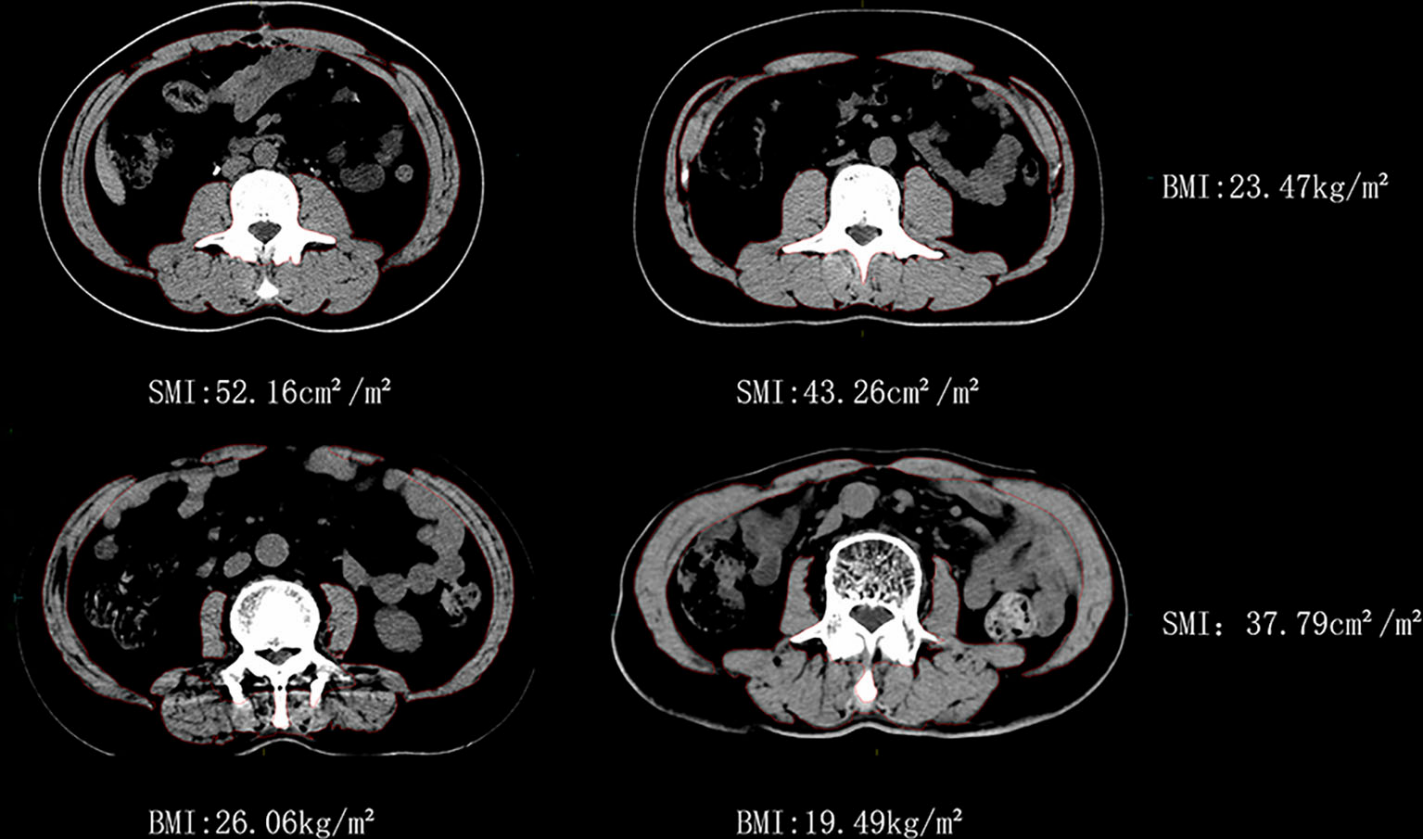
Similar BMI with different SMI vs similar SMI with different BMI for man patients.

Significant differences in BMI and SMI were observed between specific individuals, as shown in **Figure 2** (all were male patients). Two patients, C1 and C2, had similar BMI, but their SMI differed by 17.1%. The other two patients, D1 and D2, had similar SMI, but their BMI differed by 25.1%.

### 3.3 Dynamic changes in skeletal muscle index before and after chemotherapy

The dynamic changes in SMI before and after chemotherapy were further analyzed in all 38 patients, who were divided into the chemotherapy baseline group G1 and the chemotherapy completion group G2 (**Table 3**). The SMI was reduced with the completion of chemotherapy compared with the baseline chemotherapy group, regardless of gender, tumor site, adjuvant/palliative chemotherapy, chemotherapy regimen, and nutrition intervention. Notably, in the subgroup analysis with tumor site, the SMI of patients with pancreatic cancer increased by 3.38% ± 6.58% with the completion of chemotherapy compared with that at baseline. Of these 4 pancreatic cancer patients, 3 were treated with palliative chemotherapy (all chemotherapy regimens were albumin paclitaxel combined with gemcitabine), 2 males,57 years,without initial weight,1 female,69 years, with initial weight. And 1 patient received postoperative adjuvant chemotherapy (gemcitabine combined with capecitabine):female, 60 years, without initial weight.

**Table 3:** Change in SMI according to different clinical characteristics (cm^2^/m^2^).

	G1	G2	Percentage decrease (%)	Absolute value decrease	*P*
**Gender**					
Man	40.11 ± 6.54	39.22 ± 5.78	1.65 ± 8.27	0.90 ± 3.45	0.181
Female	35.02 ± 7.38	34.76 ± 7.72	0.72 ± 9.50	0.26 ± 2.77	0.774
**Tumour location**					
Oesophagus & Stomach	38.81 ± 7.12	37.89 ± 7.07	2.05 ± 9.21	0.91 ± 3.56	0.184
Pancreas	35.68 ± 6.47	36.61 ± 4.75	-3.38 ± 6.58	-0.93 ± 2.08	0.435
Colon& Rectum	40.67 ± 7.50	39.71 ± 5.32	1.58 ± 5.37	0.96 ± 2.28	0.352
**Type of chemotherapy**					
Adjuvant chemotherapy	40.32 ± 6.82	39.36 ± 6.05	1.82 ± 7.57	0.96 ± 3.37	0.169
Palliative chemotherapy	35.81 ± 6.74	35.52 ± 6.95	0.61 ± 10.31	0.29 ± 3.12	0.743
**Chemotherapy regimen**					
SOX/XELOX/FOLFOX6	39.23 ± 7.26	38.47 ± 6.51	1.31 ± 8.02	0.76 ± 3.32	0.230
Others	37.30 ± 6.44	36.66 ± 6.88	1.72 ± 10.37	0.63 ± 3.25	0.575
**Nutritional active intervention**					
Nutritional tube	34.40 ± 7.85	33.11 ± 8.36	3.68 ± 12.88	1.29 ± 3.83	0.405
Nutrition education	39.76 ± 6.59	39.16 ± 5.65	0.89 ± 7.36	0.60 ± 3.18	0.301

### 3.4 Changes in skeletal muscle-related parameters

Patients were classified into three categories according to the changes in SMI before and after chemotherapy: (1) patients with an SMI loss of >2% were defined as having skeletal muscle loss, (2) patients with an SMI increase of >2% were defined as having skeletal muscle increase; and (3) patients with an SMI change of ≤2% were defined as having skeletal muscle stability (17).

**Figure 3:**
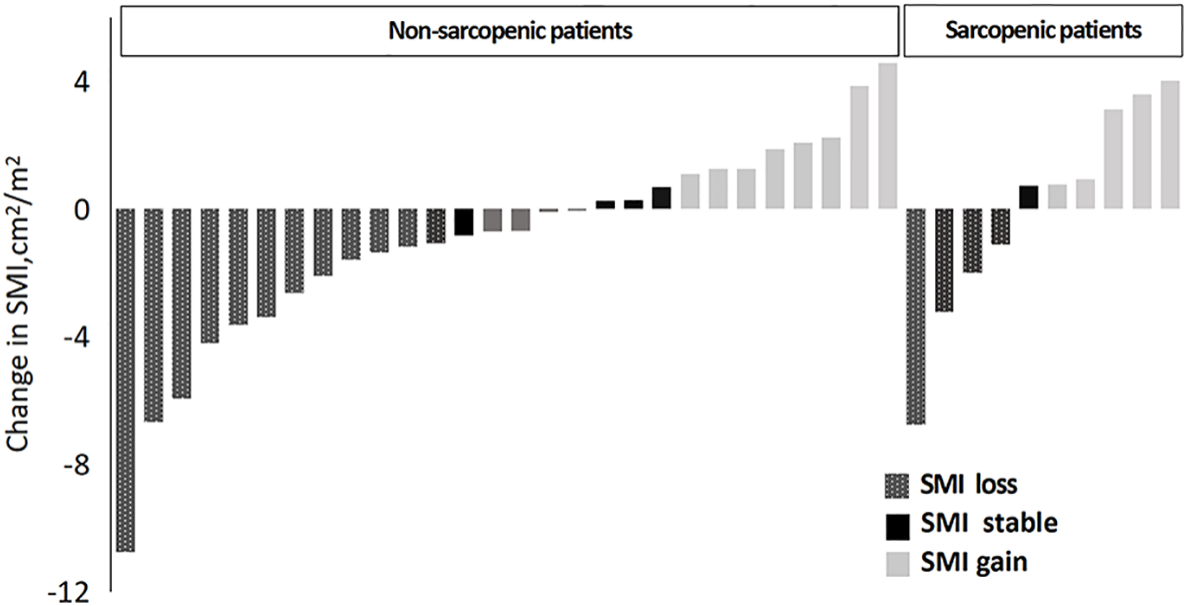
Change in skeletal muscle index in patients according to sarcopenia.

Based on the above-mentioned sarcopenia thresholds (male: L3SMI<36 cm^2^/m^2^, female: L3SMI<29 cm^2^/m^2^), the 38 patients were divided into the sarcopenic group and non-sarcopenic group according to SMI before chemotherapy. This is illustrated in **Figure 3**. The incidence of sarcopenia was reduced from 26.3% before chemotherapy to 21.1% after chemotherapy (two patients changed from the sarcopenic group before chemotherapy to the non-sarcopenic group after chemotherapy. They were both male gastric cancer patients after surgery with SOX chemotherapy, aged 47 and 69 respectively, and had a high degree of cooperation with nutrition education).

The average SMI of the entire cohort decreased from 38.77 cm^2^/m^2^ before chemotherapy to 38.04 cm^2^/m^2^ after chemotherapy, corresponding to a reduction of 0.66 kg in LBM and 2.20 cm^2^ in L3SMA (*P* > 0.05). Among all patients, 57.9% (22/38) showed stable or increasing SMI throughout chemotherapy with an average increase of 1.37 cm^2^/m^2^ (95% confidence interval [CI]: 0.65–2.08 cm^2^/m^2^, *P* = 0.001), whereas 42.1% (16/38) showed an average decrease in SMI of 3.61 cm^2^/m^2^ (95% CI: −2.16–−5.05 cm^2^/m^2^, *P*< 0.001). Muscle mass was stable or increased in 57.1% (16/28) of patients in the non-sarcopenic group and in 60.0% (6/10) of patients in the sarcopenia group before chemotherapy (*P* = 0.033).

### 3.5 Changes in skeletal muscle index and chemotherapy-related toxic adverse reactions during chemotherapy

Thirty-four patients were divided into two groups, namely those with maintained/increased SMI and those with decreased SMI. The associations between the initiation of the chemotherapy and the occurrence of toxic adverse reactions ≥ grade 3 throughout chemotherapy were analyzed. The details are shown in **Table 4**.

**Table 4:** Relevance between SMI change and toxicity reactions.

	SMI stable/gain (n=22)	SMI loss (n=16)	*P*
**Baseline status**	4 (18.2%)	6 (37.5%)	0.190
**Whole process**	12 (54.5%)	13 (81.3%)	0.089

### 3.6 Dynamic changes in L3 skeletal muscle index in patients with nutrition education or tube-feeding

**Figure 4:**
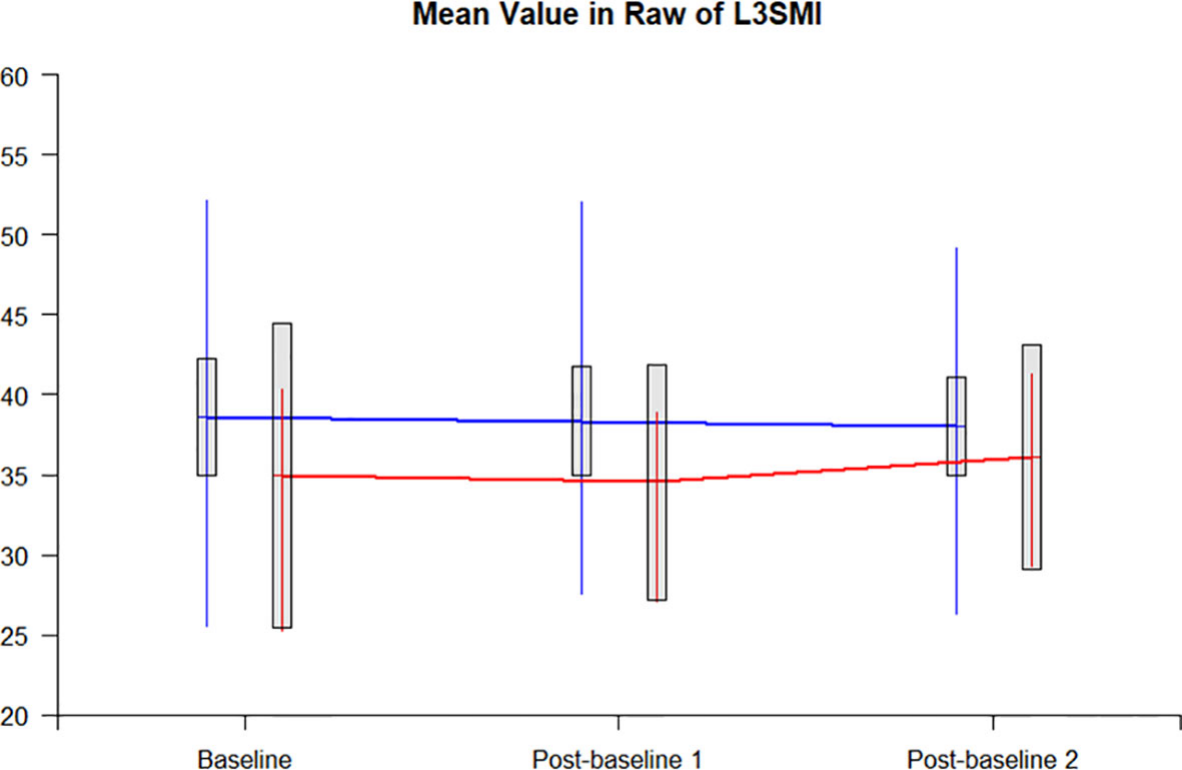
The trend of dynamic changes.

The L3SMI dynamic assessments were completed by 20 patients at baseline, during chemotherapy, and after chemotherapy. In 17 patients with nutrition education, the L3SMI decreased by 0.54 from the baseline to the completion of chemotherapy, while in 3 patients with tube-feeding, the L3SMI correspondingly increased by 1.15. The difference in the changes in L3SMI was not statistically significant between the two groups (P = 0.4973). **Figure 4** represents the trend of the dynamic changes between those with nutrition education and tube-feeding nutrition.

## 4 Discussion

Chemotherapy, as one of the main modalities of antitumor therapy, has an active and effective antitumor effect that can fundamentally reverse the uncontrollable myostatin breakdown caused by tumor growth. However, chemotherapy mechanistically causes mitochondrial injury and affects energy and myofiber metabolism. At the same time, chemotherapy increases the reactive oxygen species and induces oxidative stress in muscle, up-regulating muscle growth inhibitors and altering the balance of muscle catabolism. Chemotherapy may also lead to a reduction in muscle microvasculature through anti-angiogenesis (18–20). Moreover, chemotherapy-related toxic reactions may negatively affect food intake and physical activity, further leading to a drastic decrease in muscle mass and quantity (21). Objectively, the influence and effect of these factors on a patient’s nutritional status is dynamic. Relevant clinical studies have confirmed that the changes in L3SMI during chemotherapy (rather than the nutritional status before or after chemotherapy) are closely correlated with the recurrence-free and overall survival rates in patients with cancer (9, 22). The present study was the first prospective clinical investigation of the effects of chemotherapy on the changes in L3SMI in a Chinese population in the context of an active nutrition intervention.

The present study was initiated in September 2019 and was conducted during the outbreak of the COVID-19 pandemic, which resulted in more patient drop-outs during the follow-up and completion of only 38 cases (23).

The evaluation criteria for sarcopenia in Asian patients with cancer were adopted in the present study as L3SMI<36 cm^2^/m^2^ for males and L3SMI<29 cm^2^/m^2^ for females (16). According to these criteria, the incidence of sarcopenia in the patients enrolled in the present study was 26.3% (10/38). While according to the widely adopted Canadian criteria for sarcopenia by L3SMI (<43.0 cm^2^/m^2^ for males and<41.0 cm^2^/m^2^ for females) by Martin et al. (24), the proportion of patients with sarcopenia in the enrolled patients was 68.4% (26/38), including 8 out of 10 female patients diagnosed with sarcopenia, which failed to correspond to the actual observed situation of the patients. These results suggest that racial differences should be fully considered when using L3SMI for diagnosing sarcopenia and conducting related clinical investigations.

After six cycles of chemotherapy, L3SMI decreased from 38.77 cm^2^/m^2^ at baseline to 38.04 cm^2^/m^2^, a reduction of less than 1 cm^2^/m^2^, which was equivalent to a reduction of 0.66 kg of LBM and 2.20 cm^2^ of L3SMA (*P* > 0.05). With reference to the error criterion of 2% L3SMI measured by CT, the overall changes in skeletal muscle volume before and after chemotherapy in the present group of patients were stable (17). This was consistent with the trend of the lower NRS 2002 and PG-SGA scores after chemotherapy (*P* = 0.038 and *P* = 0.151, respectively), suggesting that active nutrition intervention reduced nutritional risk during chemotherapy and improved patients’ nutritional status.

It was noteworthy that the FFMI measurement based on BIA increased 0.69% ± 5.28% after chemotherapy. Although the difference was not statistically significant, it also indicated that the fat-free mass remained stable, with a tendency for further improvement under active nutrition intervention.

The reduction in L3SMI after chemotherapy in patients with gastroesophageal cancer in the present study was 2.05% ± 9.21%, which was significantly lower than a reduction in L3SMI of 11.30% ± 13.00% observed in patients with advanced gastric cancer receiving palliative chemotherapy by Park et al. in 2020 and a reduction in L3SMI of 6.20% ± 6.80% with adjuvant chemotherapy for gastric cancer reported by Yamaoka et al. (10, 25) in Japan in 2015.

In the present study, there was an absolute increase in L3SMI of 0.93 ± 2.08 cm^2^/m^2^ after chemotherapy in patients with pancreatic cancer treated with chemotherapy, which was also different from a previous retrospective analysis of a reduction in L3SMI of 3.0 ± 8.00 cm^2^/m^2^ during chemotherapy for pancreatic cancer (26).

The reduction in L3SMI of 0.96 ± 2.28 cm^2^/m^2^ during chemotherapy in patients with colorectal cancer was consistent with a reported reduction in L3SMI of 0.52 cm^2^/m^2^ during chemotherapy in patients with advanced colorectal cancer and the trend of decreasing muscle mass during adjuvant chemotherapy in colon cancer (22, 27, 28). Although the enrolled patients with colorectal cancer were all non-sarcopenic, it was observed that chemotherapy still had a negative effect on muscle mass in this population, with an average reduction in SMI of 1.58% ± 5.37%. Although the FFMI measurement based on BIA increased by 0.47% ± 5.01% (*P* = 0.952), FP also increased by 0.82% ± 11.47% after chemotherapy (*P* = 0.660), suggesting that sarcopenic obesity would be a clinical issue to consider and may be an independent predictor of survival in patients with lung or colorectal cancer (24).

The incidence of sarcopenia was reduced from 26.3% before chemotherapy to 21.1% after chemotherapy in patients enrolled in the present study (two patients changed from the sarcopenia group before chemotherapy to the non-sarcopenia group after chemotherapy). Using 2% as an assessment criterion for change, 57.9% (22/38) of patients had a stable or increasing L3SMI throughout chemotherapy, with an average increase of 1.37 cm^2^/m^2^ (*P* = 0.001), while L3SMI decreased in 42.1% (16/38) of patients, with an average reduction of 3.61 cm^2^/m^2^ (*P*< 0.001). In the present study, the percentage of patients with stable or increasing L3SMI in the context of active nutrition intervention was also 57.9% (22/38), which was significantly higher than the proportion of patients with stable or increasing L3SMI of 45.7% (16/35) reported in a previous study including 35 patients with advanced lung cancer treated with first-line chemotherapy (17).

Of all the enrolled patients, 18.4% (7/38) received chemotherapy with tube-feeding enteral nutrition support. The L3SMI in this subgroup decreased from 34.40 ± 7.85 cm^2^/m^2^ before chemotherapy to 33.11 ± 8.36 cm^2^/m^2^ after chemotherapy with a reduction of 2.68% ± 12.88%. This achieved better overall control of the treatment, and two of the inoperable patients were converted to operable ones.

The relationship between chemotherapy-related toxic adverse reactions and sarcopenia was also analyzed. Data in the present study showed that the incidence of chemotherapy-related toxic adverse reactions of ≥ grade 3 was significantly higher in the sarcopenic group than in the non-sarcopenic group at baseline (60.0% vs. 14.3%) and throughout chemotherapy (100% vs. 53.6%). The incidence of chemotherapy-related toxic adverse reactions of ≥ grade 3 was significantly higher in the muscle loss group than in the muscle maintenance/increase group at baseline (37.5% vs. 18.2%) and throughout chemotherapy (81.3% vs. 54.5%). There was a trend toward a higher incidence of chemotherapy-related toxic adverse reactions of ≥ grade 3 throughout chemotherapy in the muscle loss group compared with the muscle maintenance/increase group (81.3% vs. 54.5%, *P* = 0.089). The above results suggest that the incidence of chemotherapy-related toxic adverse reactions of ≥ grade 3 was lower in the muscle maintenance/increase group because of active nutrition intervention.

There were some limitations of the present study. First, although the present study was a prospective interventional study, the number of enrolled patients was still small. Second, the patients included were not diagnosed with a single type of gastrointestinal malignancy but with a mixture of gastric, pancreatic, and colorectal cancers. Although active nutrition intervention was conducted in the present study, there was a lack of effective web-based follow-up during the out-of-hospital follow-up period for these patients. Moreover, exercise education was not conducted in the present study, which is also important for improving sarcopenia.

## 5 Conclusion

In conclusion, it was confirmed in the present prospective clinical investigation that active nutrition intervention during chemotherapy probably could decrease the degree of L3SMI reduction and the incidence of sarcopenia in patients with gastrointestinal malignancies. This would increase the proportion of patients with stable or increased skeletal muscle mass throughout chemotherapy. For active nutrition intervention in patients with malignancies, the benefit was greater in the sarcopenic group than in the non-sarcopenic group. Moreover, the incidence of severe chemotherapy-related toxic adverse reactions was lower in patients with stable or increased skeletal muscle mass. In future, a combination of nutrition education and exercise management, together with the adoption of patient-initiated reporting of clinical outcomes and more effective measures, should be conducted for a population with a single tumor species or chemotherapy regimen to assess the reduction of skeletal muscle quantity/quality during chemotherapy.

## Data availability statement

The original contributions presented in the study are included in the article/supplementary material. Further inquiries can be directed to the corresponding authors.

## Ethics statement

The studies involving human participants were reviewed and approved by Ethics Committee of Ordos Central Hospital (2021-013). The patients/participants provided their written informed consent to participate in this study.

## Author contributions

Conception and design of the research: QL and SL. Acquisition of data: TX, HL, ZL, JH, JL, GJ, and SL. Analysis and interpretation of the data: TX, QL, ZL. Obtaining financing: QL and SL. Writing of the manuscript: TX, QL, and ZL. Critical revision of the manuscript for intellectual content: TX, QL, and ZL. All authors read and approved the final draft.

## Funding

This study was supported by Ordos Health Commission Key discipline Project.

## Acknowledgments

We would like to acknowledge the hard and dedicated work of all the staff that implemented the intervention and evaluation components of the study.

## Conflict of interest

The authors declare that the research was conducted in the absence of any commercial or financial relationships that could be construed as a potential conflict of interest.

## Publisher’s note

All claims expressed in this article are solely those of the authors and do not necessarily represent those of their affiliated organizations, or those of the publisher, the editors and the reviewers. Any product that may be evaluated in this article, or claim that may be made by its manufacturer, is not guaranteed or endorsed by the publisher.
